# Conjugated linoleic acid regulates adipocyte fatty acid binding protein expression *via* peroxisome proliferator-activated receptor α signaling pathway and increases intramuscular fat content

**DOI:** 10.3389/fnut.2022.1029864

**Published:** 2022-11-29

**Authors:** Jing Chen, Ruiguo You, Yao Lv, Huimin Liu, Guoqing Yang

**Affiliations:** Laboratory of Animal Gene Engineering, College of Life Sciences, Henan Agricultural University, Zhengzhou, China

**Keywords:** intramuscular fat, conjugated linoleic acid, adipocyte fatty acid binding protein, peroxisome proliferator-activated receptor α, meat quality

## Abstract

Intramuscular fat (IMF) is correlated positively with meat tenderness, juiciness and taste that affected sensory meat quality. Conjugated linoleic acid (CLA) has been extensively researched to increase IMF content in animals, however, the regulatory mechanism remains unclear. Adipocyte fatty acid binding protein (A-FABP) gene has been proposed as candidates for IMF accretion. The purpose of this study is to explore the molecular regulatory pathways of CLA on intramuscular fat deposition. Here, our results by cell lines indicated that CLA treatment promoted the expression of A-FABP through activated the transcription factor of peroxisome proliferator-activated receptor α (PPARα). Moreover, in an animal model, we discovered that dietary supplemental with CLA significantly enhanced IMF deposition by up-regulating the mRNA and protein expression of PPARα and A-FABP in the muscle tissues of mice. In addition, our current study also demonstrated that dietary CLA increased mRNA expression of genes and enzymes involved in fatty acid synthesis and lipid metabolism the muscle tissues of mice. These findings suggest that CLA mainly increases the expression of A-FABP through PPARα signaling pathway and regulates the expression of genes and enzymes related to IMF deposition, thus increasing IMF content. These results contribute to better understanding the molecular mechanism of IMF accretion in animals for the improvement of meat quality.

## Introduction

Intramuscular fat (IMF), deposited within muscle tissue, is an important characteristics of meat quality (1, 2). IMF determines sensorial qualities of meat including flavor, tenderness, and juiciness. However, in the past few decades, lean meat yield and backfat thickness were considered as important parameters in breeding, which result in decreasing IMF content. Recently, improving IMF content of meat is a critical interest to nutritionists, breeders and geneticists for health and economic reasons. IMF deposition is the result of comprehensive effects of animal growth, body fat distribution, fatty acid composition, key genes of fat metabolism and transcription regulators (3–5). Multiple factors greatly influence IMF content, such as breeds, gender, age, genes and dietary (6–8). Conjugated linoleic acid (CLA) is a group of positional and geometric isomers of linoleic, which has a variety of biologically beneficial activities including anti-cancer, anti-obesity, anti-inflammatory, anti-diabetic and immune modulating properties (9–11). CLA has been reported to modulate body fat composition through distinct actions on fat deposition, lipolysis of adipose cells and overall lipid metabolism (12). Previous studies have shown that CLA improved IMF content and marbling scores in pigs, cattle, lamb and broiler chicken (13–18). However, there are limited studies about the mechanism of CLA on increasing IMF content.

Adipocyte fatty acid binding protein (A-FABP) is expressed in adipose tissue, interacts with peroxisome proliferators activated receptors (PPARs) and binds to hormone-sensitive lipase and therefore, plays an important role in the lipid deposition of muscles and homeostasis in adipocytes (8). The polymorphism of the A-FABP gene mutation site is positively correlated with IMF content (19, 20). Meanwhile, many studies have shown that the high expression of A-FABP gene in muscle tissue is conducive to improve the IMF content (21–23). Therefore, A-FABP can be used as a candidate gene for enhancing IMF content. A-FABP promoter contains a peroxisome proliferator response element (PPRE) binding site, and the promoter can be activated by PPARs (24–26). PPARs belong to the nuclear receptor super-family of transcription factor, which are divided into three isoforms: PPARα, PPARβ/δ and PPARγ, associated with adipogenesis, lipogenesis and IMF accretion in animals (27–29). PPARs are activated by specific natural ligands such as lipids, retinoids, steroids, and thyroid hormones. PPARs heterodimerize with retinoid X receptors (RXRs) and upon ligand activation bind to PPRE in the regulatory regions of their target genes (30). PPARs regulate genes coding for proteins participating in fatty acid uptake and mitochondrial β-oxidation. CLA regulates the expression of a variety of nuclear transcription factors and genes involved in lipid metabolism and affected the uptake and oxidation of fatty acids and lipid anabolism to increase IMF deposition and improve meat quality (31–33). A recent study revealed that dietary supplemental with CLA increased the expression of PPAR-γ and A-FABP, enhanced IMF deposition by 14%, and reduced subcutaneous fat deposition by 9.2% in Landrace and Yorkshire hybrid pigs (14). However, the underlying mechanism of genes expression related to IMF deposition by CLA regulated is yet to be elucidated.

The present study aims to investigate molecular mechanism of CLA promoting IMF deposition. In this study, in cellular level, we observed that CLA treatment enhanced the activity of PPARα and A-FABP promoter. Overexpression of PPARα in cells dramatically facilitated the activity of A-FABP promoter, while siRNA-mediated knockdown of PPARα expression decreased the activity of A-FABP promoter, which indicated PPARα maybe bind to the promoter region of A-FABP and thus regulate its transcriptional expression. Hence, we inferred that CLA activated the expression of transcription factor PPARα, which in turn regulating the transcription expression of A-FABP. Furthermore, in mouse model, our results showed that dietary supplemental with CLA increased the abundance of lipid droplets and improved the IMF content in the quadriceps femoris of mice. Additionally, the mRNA and protein expression of PPARα and A-FABP were remarkably increased in the quadriceps femoris of mice fed CLA. We further found that CLA increased IMF accumulation by regulating the expression of genes and enzymes related to fatty acid synthesis and lipid metabolism. These findings uncovered a molecular regulatory pathway of CLA-enhanced IMF deposition.

## Materials and methods

### Animals, diets, and treatments

All experiments involving animals were conducted in accordance with guidelines established by the Animal Care and Use Committee of Henan Province, China. Kunming mice aged 35 days (*n* = 40) were housed under standard conditions with 12/12-h light/dark cycles at 22 ± 2°C, 50% of humidity and had free access to water and food. Mice were fed a standard diet for 1 week for adaptation and were randomized into two groups. Twenty mice in control group were fed a basal diet (Con group) and those in the experimental groups were fed the basal diet supplemented with 1.5% CLA (CLA group). The experiment lasted for 15 days. The composition of CLA diet was showed in Supplementary Tables 1, 2.

### Sample collection

At the end of the experiment, the mice were euthanized with 1% sodium pentobarbital anesthesia. Livers and the quadriceps femoris muscles of mice were quickly sampled, weighed, and partially fixed in 4% paraformaldehyde for future analysis. Others were stored at −80°C for RNA and protein extraction.

### Plasmid constructs

DNA fragment of porcine PPARα promoter from −1069 to +143, and porcine A-FABP promoter from −1128 to +3 were amplified by PCR using porcine liver genomic DNA. For the generation of the luciferase reporter construct, the PCR products were purified and subsequently digested using *Kpn*I and *Bgl*II restriction enzymes, then cloned into the corresponding restriction sites of pGL3-basic vector using Clon Express Ultra One Step Cloning Kit (Vazyme, USA). The plasmids were named pGL3-pparα and pGL3-afabp. The PCR conditions were as follows: 94°C for 3 min, then 35 cycles of 94°C for 30 s, 65°C for 40 s and 72°C for 90 s, followed by a final extension at 72°C for 10 min. The porcine PPARα sequence (GenBank accession number:AY364466) was amplified from porcine liver by PCR. The PCR product was digested with restriction enzymes *kpnI* and *Eco*RI, and cloned into expression vector pCMV5-myc to generate pCMV5-myc-pparα. The PCR conditions were as follows: 94°C for 3 min, then 35 cycles of 94°C for 30 s, 67°C for 40 s and 72°C for 30 s, followed by a final extension at 72°C for 10 min. The primer sequences used for cloning of promoter and plasmid construction are shown in Table 1.

**TABLE 1:** Primers or oligonucleotides for PCR.

Gene (Pig)	Primers	EXP
PPARα	Fwd:CCAGCCTCCAGCCCCTCGT	PCR
	Rev:CATGACCTAGAAGATGCCGAGAC	
PPARα promoter	Fwd:GCACACGGGGAACAGATAAC	PCR
	Rev:CTTCCAGAACTGTCCTCACCAATG	
A-FABP promoter	Fwd:TGGGAAGATTTCAGGATACT	PCR
	Rev:CATTTTGTGAGCACTCTAGG	

### Cell lines and cell culture

The 293T (Human Renal Epithelial Cells), C2C12 (Mouse Myoblasts), 3T3-L1 (Mouse Preadipocytes) and PK15 (Porcine Renal Epithelial Cells) cell lines were preserved in our laboratory, and maintained in DMEM (Invitrogen, Carlsbad, CA, USA) plus 10% fetal bovine serum (Gibco, Waltham, MA, USA), 2 mM of glutamine (Gibco, Waltham, MA, USA), 50 U/mL penicillin (Gibco, Waltham, MA, USA) and 50 μg/mL of streptomycin (Gibco, Waltham, MA, USA). All cells were cultured in a 37°C incubator with 5% CO_2_.

### Peroxisome proliferator-activated receptor α knockdown analysis

For knockdown of porcine PPARα, small interfering RNA (siRNA) was purchased from GenePharma (Shanghai, China). The sequences to interfere porcine PPARα (siRNA-1 and siRNA-2) expression, and control sequences (siRNA-Con) were listed in Table 2. PK15 were transfected with siRNA-Con or porcine PPARα siRNA by Lipofectamine^®^ 3000 reagent (Thermo Fisher Scientific, USA). At 48 h after transfection, cells were used to detect mRNA and protein levels of PPARα.

**TABLE 2:** siRNA interference sequence.

Gene	Sequence	EXP
PPARα siRNA-1	Fwd:CCUAAACGUAGGACACAUUTT	interfere
	Rev:AAUGUGUCCUACGUUUAGGTT	
PPARα siRNA-2	Fwd:CCAACGGCAUCCAGAACAATT	interfere
	Rev:UUGUUCUGGAUGCCGUUGGTT	
siRNA-Con	Fwd:UUCUCCGAACGUGUCACGUTT	negative control
	Rev:ACGUGACACGUUCGGAGAATT	

### Luciferase assay

The cells were seeded at a density of 1.2 × 10^5^ cells/well in 24-well plate for 24 h, and then transfected with different plasmids (pGL3-pparα, pGL3-afabp, and pCMV5-myc-pparα) using Lipofectamine^®^ 3000 reagent. All plasmids were used in equimolar amounts, and they were co-transfected with 50 ng pRL-TK, a Renilla luciferase reporter vector as internal control. After 4 h, the cells were treated with 100 μM CLA. At 48 h after transfection, cells were harvested to measure the luciferase activity. Transfected cells were lysed with Passive Lysis Buffer (Promega), and assayed for Firefly and Renilla luciferase activities in a luminometer by the Dual-Luciferase Reporter Assay System according to the manufacturer’s instructions. The Firefly luciferase activity was normalized against Renilla luciferase activity. Results were normalized to the control vector pGL3-Basic.

The configuration of 100 μM CLA is as follows: 3.57 mL absolute ethanol was added to 100 mg CLA, from which 1 mL was blown dry with nitrogen to obtain the storage solution of CLA. 0.05 mL of the storage solution was added to 1.55 mL of 0.1M NaOH to get 100 mM solution, which was diluted 1‰ with DMEM and filtered.

### Real time quantitative PCR analysis

Total RNA from cells and muscle samples were extracted using Trizol (Takara, Dalian, China). cDNA was synthesized using the PrimeScript II 1 st Strand cDNA Synthesis Kit (Takara, Dalian, China). RT-qPCR was performed with SYBR Green PCR master mix (Takara, Dalian, China) on a 7500 Real Time PCR System (Applied Biosystems, CA, USA). The program was as follows: 95°C for 5 min, followed by 40 amplification cycles, each at 95°C for 10 s, then 60°C for 30 s. The glyceraldehyde-3-phosphate dehydrogenase (GAPDH) gene was used as a reference gene for the standardization of the results. The data was analyzed using the cycle threshold (2^–△△CT^) method. Primer sequences for target genes were listed in Table 3.

**TABLE 3:** Primers or oligonucleotides for real-time PCR (mouse or pig).

Gene	Primers	EXP
CPT1	Fwd:TCAAGCCAGACGAAGAACA	Real-time
	Rev:GCACCTTCAGCGAGTAGCG	PCR
AMPK	Fwd:ACCATACCCATAGGATTGAC	Real-time
	Rev:CATAGGGATTTGTTGCTCTT	PCR
ACOX1	Fwd:ATCACCATCCCAGGAGTA	Real-time
	Rev:TAGAAGGCTTAGGCAACA	PCR
ACOX3	Fwd:CGCTGGCTTGTTTGCTACT	Real-time
	Rev:CTGGCTGTTGTTTCTTGCTTC	PCR
LCAD	Fwd:GGCCCTTGATAAATCCTTT	Real-time
	Rev:TGATCTCGTGATCGTCGTG	PCR
CD36	Fwd:GAGGCGGGCATAGTATCA	Real-time
	Rev:GGCAGGAGTGCTGGATTA	PCR
FAS	Fwd:CCATCGCTTCCAGGACAAT	Real-time
	Rev:GGCTTCGCCAACTCTACCA	PCR
LACS	Fwd:CAGGTCGCAGATAGATGAAC	Real-time
	Rev:ATTGGTACGAGGAGGATTGT	PCR
DGAT1	Fwd:TAGGCTTGTAGAAGTGTCTGATG	Real-time
	Rev:GAGATTGGTGGAATGCTGAG	PCR
ACC	Fwd:AAGGCAGTATCCATTCATCACA	Real-time
	Rev:ACACGGGCAGTCTACCACAG	PCR
A-FABP	Fwd:GATGAAATCACCGCAGACGACA	Real-time
	Rev:ATTGTGGTCGACTTTCCATCCC	PCR
PPARα	Fwd:AGTGCCTGTCTGTCGGGATG	Real-time
	Rev:CTCTTGCCCAGAGATTTGAGGTC	PCR
β-actin	Fwd:GCTCTGGCTCCTAGCACCAT	Real-time
	Rev:GCCACCGATCCACACAGAGT	PCR
PPARα (pig)	Fwd:TCAAGAGCCTGAGGAAACC	Real-time
	Rev:CAAATGATAGCAGCCACAAA	PCR
GAPDH(pig)	Fwd:CACAGTCAAGGCGGAGAACG	Real-time
	Rev:CCATTTGATGTTGGCGGGAT	PCR

### Intramuscular fat content analysis

The IMF was measured by the Soxhlet extractor method according to GB5009.6-2016. The muscle samples were cut into thin slices, and put into glassware, and dried at 105°C for more than 13 h to absolute dry. After crushing, 5 g was weighed and wrapped with quantitative filter paper, which dried at 105°C for at least 2 h until its weight did not change, and the dried paper bag (x) was weighed. The dried paper bag was put into a Soxhlet extraction bottle and refluxed at 65°C by an ether reflux device. When the drip was transparent, the paper bag was taken out, distributed in a clean enamel plate, placed in a ventilating cabinet for 30 min, dried at 105°C for more than 2 h to fully volatilize the ether until its weight did not change, and weighed (y). Each sample was repeated for 3 times. The IMF content was calculated according to formula: IMF content (%) = (x-y)/a × 100.

### Oil Red O staining analysis

The liver and quadriceps femoris muscles of mice were fixed with 4% formaldehyde, thereafter made into frozen slices. Slides were dried, fixed in stationary liquid for 15 min and then washed in distilled water for 30 s. Afterward, slides were dried and then incubated with Oil-Red-O working solution for 10 min. Then slides were washed with in distilled water and 60% isopropanol, and then incubated with hematoxylin solution for 5 min, washed in running water for 5 min, mounted using glycerol jelly mounting medium. Images were captured using an a fluorescence microscope.

### Western blotting analysis

The cells or quadriceps femoris muscles of mice were lysed with RIPA lysis buffer supplemented with 1% protease inhibitor cocktail. Equivalent amounts of supernatant samples were run on 10% acrylamide SDS-PAGE gel. Then the protein was transferred to nitrocellulose membrane (Millipore, Billerica, MA, USA). Membranes were blocked with 5% milk for 1 h and incubated with antibodies against PPARα (1:2000) (Cell Signaling Technology, Boston, MA), A-FABP (1:2000) (Cell Signaling Technology, Boston, MA) or β-actin (1:2000) (Cell Signaling Technology, Boston, MA) at 4°C overnight. Next, membranes were incubated with HRP-conjugated secondary antibody (1:3000) (Cell Signaling Technology, Boston, MA) for 1 h at room temperature. The protein bands were visualized with ECL detection kit (Cell Signaling Technology, Boston, MA). Densitometry analysis was performed using Image J software.

### Statistical analysis

GraphPad Prism 8.0 was used for data management. The data were expressed as the means ± SEM. Statistical differences between experimental and control groups were evaluated by Student’s *t*-test and statistical significances were indicated as follows: ^***^p < 0.001; ^**^p < 0.01; *p < 0.05.

## Results

### Conjugated linoleic acid enhanced the activity of peroxisome proliferator-activated receptor α and adipocyte fatty acid binding protein promoters

**FIGURE 1:**
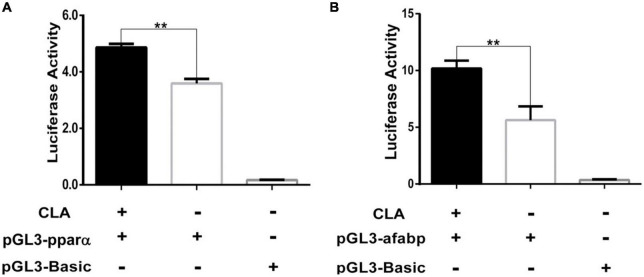
Conjugated linoleic acid (CLA) enhanced the activity of PPARα and A-FABP promoter. **(A,B)** The 293T cells was plated in 24-well plates and then transfected with pGL3-pparα, or pGL3-afabp, and pGL3-Basic, pRL-TK. After 4 h, the cells were treated with CLA at a 100 μM final concentration. Dual luciferase reporter gene system was used to measure the activity of PPARα and A-FABP promoter. Data were analyzed by *t*-test. **P < 0.01.

A recent research has reported CLA can bind PPAR subtypes to regulate a series of genes expression (29). To investigate the regulation effect of CLA on porcine PPARα and A-FABP genes, the promoter sequences of porcine PPARα and A-FABP were cloned by PCR and verified by DNA sequencing (Supplementary Figure 1). According to CLA dose and time test (Supplementary Figures 2A,B), 100 μM CLA was chosen in the subsequent experiment. The luciferase reporter plasmids containing the PPARα promoter region (pGL3-pparα) or containing the A-FABP promoter region (pGL3-afsbp) were transfected into 293T cells, and then the cells were treated with 100 μM CLA. We found that CLA significantly enhanced the activity of the porcine PPARα (Figure 1A) and A-FABP promoter (Figure 1B).

### Peroxisome proliferator-activated receptor α increased the activity of adipocyte fatty acid binding protein promoter

**FIGURE 2:**
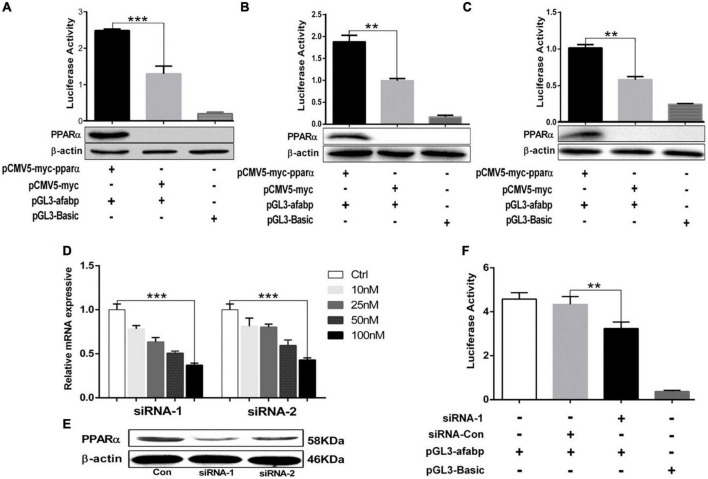
PPARα increased the activity of A-FABP promoter. **(A–C)** The 293T **(A)**, C2C12 **(B)** and 3T3-L1 **(C)** cell were co-transfected with pGL3-afabp, pCMV5-myc-pparα, pGL3-Basic, pRL-TK or pCMV5-myc for 48 h. Dual luciferase reporter gene system was used to detect the activity of A-FABP promoter (upper panel), and western blotting was performed to detect PPARα expression (lower panel). PK15 cells were transfected with the indicated siRNAs targeting porcine PPARα at different concentrations (10, 25, 50, and 100 μM). RT-qPCR **(D)** and western blotting **(E)** were performed to detect the expression level of PPARα, and GAPDH was used as an internal reference gene. **(F)** PK15 cells were transfected with pGL3-afabp or pGL3-Basic for 24 h and then treated with siRNA-1 or siRNA-Con as indicated. Dual luciferase reporter gene system was used to analysis the activity of the A-FABP promoter. Data were analyzed by *t*-test. **P < 0.01, ***P < 0.001.

**FIGURE 3:**
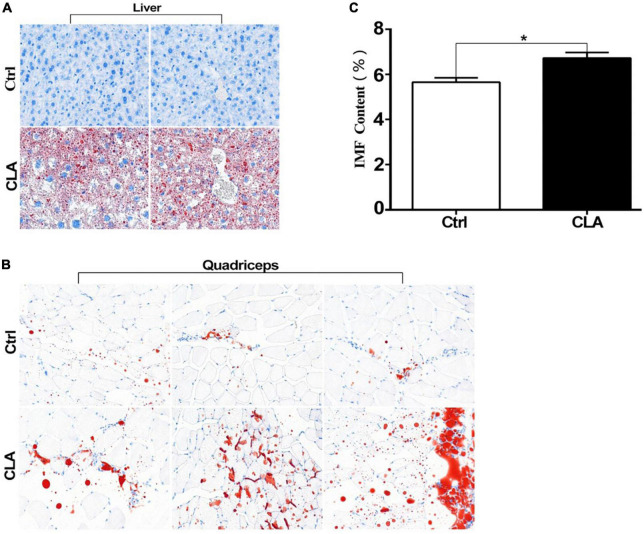
Conjugated linoleic acid promoted fat deposition in liver and the quadriceps femoris of mice. **(A,B)** The liver tissues and quadriceps femoris muscles of mice were fixed with 4% formaldehyde. Frozen slides were made from these tissues and then stained with oil red O (magnification:200X). **(C)** The IMF content in the quadriceps femoris of mice was determined by the Soxhlet extraction method. Data in panel **(C)** were analyzed by *t*-test. *P < 0.05.

A-FABP contains PPRE sites in its promoter region and can be regulated by PPARs (24, 26). To further verify the potential effect of PPARα on the transcriptional regulation of A-FABP gene, we constructed a PPARα eukaryotic expression plasmid (pCMV5-myc-pparα). The plasmids pCMV5-myc-pparα and pGL3-afabp were co-transfected into 293T cells (Figure 2A), C2C12 cells (Figure 2B) and 3T3-L1 cells (Figure 2C). The results indicated that PPARα significantly increased the activity of porcine A-FABP promoter. In addition, the over-expression of PPARα in 293T, C2C12 and 3T3-L1 cell was detected by western blotting. PPARα-specific siRNAs treatment efficiently reduced the expression of PPARα, and the inhibition efficiency of two candidate siRNAs (siRNA-1and siRNA-2) was 60 and 50%, respectively (Figures 2D,E). The most effective siRNA, siRNA-1, was used to silence the expression of porcine PPARα in subsequent trials. SiRNA-mediated knockdown of PPARα expression reduced the activity of porcine A-FABP promoter in PK15 cells (Figure 2F). Taken together, these data suggest that PPARα may be bind to the promoter region of A-FABP, thus regulating its transcriptional expression.

### Dietary conjugated linoleic acid promoted intramuscular fat deposition in the quadriceps femoris of mice

To explore the effect of CLA on regulating fat deposition at animal level, 5-week-old Kunming mice were randomly divided into a control group and an experimental group. The experimental group was fed a diet containing CLA, and the control group was fed a basal diet. CLA supplementation has no effect on body weight changes of mice (Supplementary Figure 3). As shown in Figure 3A, compared to the control group, abundant large of red lipid droplets in the livers of mice fed with CLA was significantly increased by Oil Red O staining. According to fat droplets in liver, the mouse model of dietary CLA addition in our study was successfully established. The IMF content in the quadriceps femoris of mice was determined by the Soxhlet extractor method. As shown in Figure 3C, dietary CLA addition significantly increased IMF content in the quadriceps femoris of mice compared with the control group. In addition, we observed that a large number of red lipid drops in the quadriceps femoris cells of mice compared with the control group (Figure 3B). These results showed that CLA significantly increased the abundance of lipid drops in the quadriceps femoris cells of mice, thus promoting IMF deposition. Collectively, these data indicate that dietary CLA has a general effect on the IMF deposition in mice.

### Dietary conjugated linoleic acid upregulated the expression of peroxisome proliferator-activated receptor α and adipocyte fatty acid binding protein in the quadriceps femoris of mice

**FIGURE 4:**
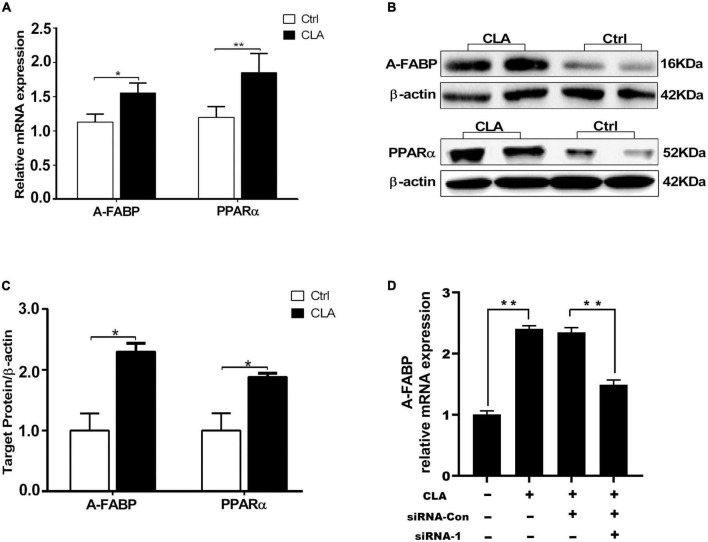
Dietary CLA up-regulated A-FABP and PPARα expression in the quadriceps femoris of mice. **(A–C)** The total RNA from the quadriceps femoris of mice was extracted and RT-qPCR was performed to detect the mRNA levels of A-FABP and PPARα **(A)**, and GAPDH was used as internal reference gene. RT-qPCR was analyzed using the cycle threshold (2^–△△CT^) method. Western blotting was performed to measure the protein level of A-FABP and PPARα **(B)**, and the relative level of A-FABP and PPARα was analyzed by Image J **(C)**. **(D)** SiRNA control (siRNA-Con) or siRNA-mediated knockdown of PPARα expression (siRNA-1) was transfected into 3T3-L1 cells for 4 h, and was then stimulated with CLA (100 μM) for 48 h. The A-FABP mRNA levels were measured by RT-qPCR. Data in panels **(A,C,D)** were analyzed by *t*-test. *P < 0.05, **P < 0.01.

Conjugated linoleic acid promotes IMF deposition and improves meat quality by increasing the expression of specific genes in intramuscular adipose tissue (16). In cellular level, our results showed that CLA enhanced PPARα and A-FABP promoter activity. Therefore, we analyzed the expression level of PPARα and A-FABP in the quadriceps femoris of mice. RT-qPCR results showed that dietary CLA addition significantly increased the mRNA levels of PPARα and A-FABP in the quadriceps femoris of mice (Figure 4A). Western blotting results showed that dietary CLA addition also increased the protein levels of PPARα and A-FABP in the quadriceps femoris of mice (Figure 4B). The band intensities were quantified using ImageJ 1.42q software (Figure 4C). These results demonstrated that dietary supplementation with CLA increased the expression of PPARα and A-FABP in the quadriceps femoris of mice. To more fully explore CLA-induced A-FABP expression is regulated by PPARα, siRNA-mediated knockdown of PPARα expression (siRNA-1) or siRNA control (siRNA-Con) was transfected into 3T3-L1 cells in the presence and absence of CLA. We found that A-FABP expression was significantly increased upon treatment with CLA, while A-FABP expression was greatly reduced in response to si-PPARα (Figure 4D). These results indicated that CLA-induced A-FABP expression was regulated by PPARα in 3T3-L1 cells. Combine the front results, we concluded that CLA may be increase the expression of A-FABP by binding and activating PPARα promoter, and then the high expression of A-FABP increase fat deposition and IMF content.

### Dietary conjugated linoleic acid regulated the expression of genes and enzymes involved in fatty acid synthesis and lipid metabolism in the quadriceps femoris of mice

**FIGURE 5:**
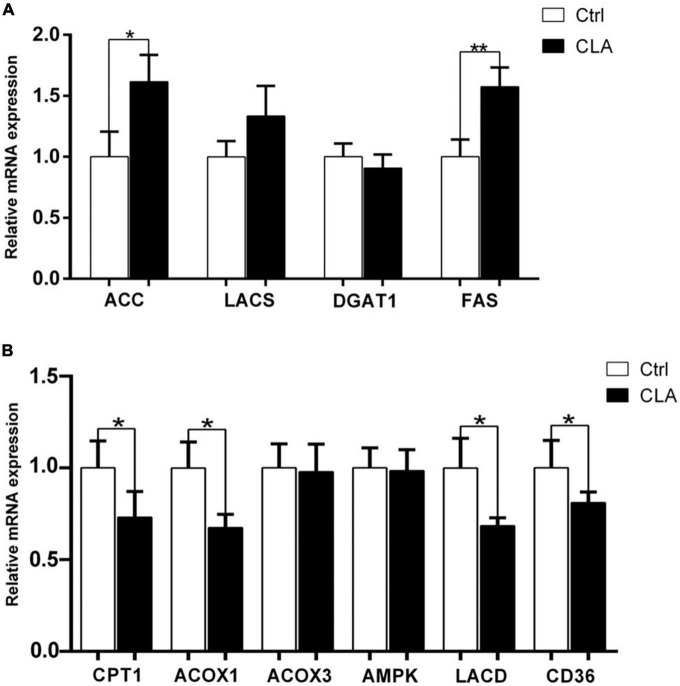
Dietary CLA regulated the expression of genes involved in fatty acid synthesis and lipid metabolism in the quadriceps femoris of mice. **(A,B)** RT-qPCR was performed to determine the expression levels of genes related to fatty acid synthesis (ACC, LACS, DGAT1, and FAS) and lipid metabolism (CPT1, ACOX1, ACOX3, AMPK, LCAD, and CD36) in the quadriceps femoris of mice. The expression levels were normalized to GAPDH mRNA levels. Data were analyzed by *t*-test. *P < 0.05.

The IMF content depends on the synthesis, transport and deposition of fatty acids. Different studies have reported many genes and enzymes related to fatty acid synthesis and lipid metabolism showed to be the key drivers of the observable increase in IMF content in animals (34, 35). The mRNA levels of genes related to fatty acid synthesis were showed in Figure 5A. The mRNA levels of acetyl- CoA carboxylase (ACC) and fatty acid synthase (FAS) mRNAs were significantly increased, while the mRNA levels of long-chain acyl-CoA synthetase (LACS) and diacylglycerol acyltransferase (DGAT) were no significant difference between the control and the CLA group. The mRNA levels of genes related to lipid metabolism were showed in Figure 5B. The mRNA levels of carnitine palmitoyltransferase-1 (CPT-1), acyl-CoA oxidase-1 (ACOX1), long chain acyl-CoA dehydrogenase (LCAD) and fatty acid translocase (FAT/CD36) mRNA levels were significantly decreased, while the mRNA levels of acyl-CoA oxidase-3 (ACOX3) and adenosine-monophosphate-activated protein kinase (AMPK) were no significant difference between the control and the CLA group. These results indicated that dietary CLA may increase IMF deposition by modulating the expression of fatty acid synthesis and lipid metabolism related genes.

## Discussion

Intramuscular fat is an important economic trait for meat quality. Much attention has been paid to the augmenting of the IMF content to satisfy the eating experience of the consumer. In this study, our results demonstrate that the treatment of CLA in cells enhanced the activity of the PPARα promoter, and PPARα played an important role in regulating the transcriptional expression of A-FABP gene. The results of animal experiments showed that dietary CLA addition increased the abundance of lipid droplets and increased the IMF content in the quadriceps femoris of mice. Additionally, we found that both the PPARα and A-FABP mRNA and protein expression were consistent with IMF content. In this study, we confirmed that CLA affects the IMF content by regulating the expression of A-FABP through PPARα signaling pathway.

Previous studies found that the dietary CLA supplementation significantly improved IMF content, and A-FABP mRNA expression was significantly and positively correlated with IMF deposition (18). However, there is unclear the mechanism by which CLA regulates A-FABP expression. Studies have confirmed that the binding of PPARγ to Long Chain Fatty Acids (LCFAs) increased its concentration, which induced and regulated A-FABP expression (36). Additionally, previous research showed PPARs agonists increased A-FABP expression in pT1 tumors to prevent cancer progression (37). A-FABP is known to contain PPRE sites in its upstream promoter region. PPARs was believed to recognize the PPRE elements located in the promoter region of target genes. PPARα binds to the consensus sequence PPRE (5′-AACTAGGACA (N) AGGTCA-3′) in the promoter region of the rat acyl-CoA oxidase (AOX) gene by a series of mutational analyses. Our results demonstrated that ectopic expression PPARα significantly increased the activity of A-FABP promoter, while knockdown of PPARα expression reduced the activity of A-FABP promoter. These results indicated that PPARα regulated the transcriptional expression of A-FABP. Thus, we inferred that PPARα played an important role in regulating A-FABP expression. However, it needs to be explored the molecular mechanism of PPARα regulating A-FABP expression in further.

Previous research has shown that CLA directly or indirectly affected the PPARγ ligand to regulate PPARγ expression, thereby regulating A-FABP expression, improving the adipogenic differentiation ability of cells, and accelerating the deposition of lipid drops (38, 39). CLA increased the liver fatty acid oxidation capacity by activating PPARα (40, 41). CLA induced the expression of fibroblast growth factor 21 (FGF21) by activating PPARα in the liver, thus regulating liver lipid metabolism (42). CLA activated PPARα, thereby increasing the expression and activity of CPT-1 and promoting the decomposition of triglycerides in subcutaneous fat and fatty acid oxidation, which reduces subcutaneous fat deposition (18). In our research, we found that CLA significantly enhanced the activity of PPARα promoter and promoted PPARα expression. One study demonstrated that CLA significantly increased PPARα gene expression in skeletal muscle (43), which was consistent with our studies. We conjectured that CLA promoted the PPARα expression by enhancing the activity of PPARα promoter, and PPARα enhanced the activity of A-FABP promoter and increased A-FABP expression. There is a positive feedback regulation mechanism. In any case, the important mechanism of CLA-mediated the regulation of the A-FABP expression through the PPARα signaling pathway is worthy of further exploration.

Dietary intervention is one of the most common methods to improve the IMF content of animals. Oregano essential oil (OEO) supplementation to a reduced-protein, amino acid-supplemented diets improved the IMF content (44). Recent studies have demonstrated that the higher energy level of the diets increase IMF deposition (45). Several studies have shown that supplementation of barley, Betaine, L-arginine to diets of pigs increase marbling score and IMF content (46–48). Supplementation of CLA to diets also has been reported to enhance marbling score and IMF content in pigs (49, 50). Our results are consistent with these findings that dietary CLA improved IMF deposition in animal experiments. Conversely, some researches have reported that there is a decrease in IMF content of CLA fed pigs (51, 52). In consistencies of these results might be contributed to the source of CLA, the abundance of fat in dietary, sex, breed and percentage of lean and duration of the feeding program (53). In addition, our current study also demonstrates that dietary CLA supplementation upregulates the expression of A-FABP gene in the quadriceps femoris of mice. In agreement, several studies have identified that A-FABP is a candidate gene of IMF content, due to its functional role in fatty acid transport as well as fat deposition by regulating lipid metabolism-related genes (54–56). PPARα is highly expressed in the liver, heart, muscle tissue, etc., which has an important function in fatty acid catabolism (57). Recent study has revealed that the activation of PPARα induced the upregulation of fatty acid transport and β-oxidation (58). Over-expressing PPARα has been found an increasing fatty acid up-take and oxidation in muscle of mice (59). Currently, in our research, we discovered that dietary CLA significantly increased PPARα expression in the quadriceps femoris of mice. However, previous studies reported that PPARα was involved in subcutaneous fat oxidation (60). The possible reason is that PPARα not only has the function of increasing fatty acid uptake and oxidation, but also plays an important role in regulating the production of fatty acids, such as affecting the expression and activity of A-FABP.

CLA improved IMF accumulation by regulating the expression of related genes in in fat metabolism and IMF deposition. It is well known that FAS is identified as a key multifunctional enzyme involved in lipogenesis (61–63). Previous studies have reported that higher mRNA abundance of FAS regulated fatty acid synthesis in skeletal muscle. ACC, a crucial rate-limiting enzyme, catalyzes the first step in *de novo* fatty acid synthesis, resulting in the biosynthesis of long-chain fatty acids (64). Some studies have indicated that the expression of FAS and ACC was positively correlated with IMF content in mammals fed CLA (65, 66). We also observed an increase mRNA levels of FAS and ACC in the quadriceps femoris of mice fed CLA. This finding was consistent with recent researches, which revealed that CLA accelerated the capacity of fatty acid synthesis by up-regulating the expression levels of fatty acid synthesis related genes, leading to increase IMF accumulation. In addition, lipid metabolism participated in IMF deposition. CPT-1 is an essential rate-limiting enzyme involved in fatty acid metabolism. CPT-1 has been identified to transport esters of fatty acids to mitochondria for β-oxidation, thus implying changes in mitochondrial fatty acid oxidation (67). In this study, the mRNA levels of CPT-1 were decreased in the quadriceps femoris of mice fed CLA, which suggested that fatty acid oxidation in muscle was reduced. ACOX1 is the rate-limiting enzyme in peroxisomal fatty acid β oxidation pathway (68). ACOX1 has been identified to involve in lipid metabolism and fat deposition in mice, pigs, and fish (69–71). Our results were in agreement with previous data showing ACOX1 down-regulated by dietary CLA in mice (72). LCAD, a rate-limiting enzyme, catalyzes the first-step reaction of mitochondrial fatty acid β oxidation, thus involving in fatty acid oxidation (73, 74). CD36 has been identified to facilitate and modulate the uptake of fatty acids. Notably, CD36 acts a mitochondrial membrane protein that regulates mitochondrial fatty acid uptake and oxidation (75–77). Our results showed that CPT-1, ACOX1, LCAD and CD36 were significantly decreased in the quadriceps femoris of mice fed CLA, which suggested that CLA may reduce fatty acid oxidation, thus leading to increase IMF accumulation. Based on these data, we speculated that dietary CLA supplementation may increase IMF deposition by regulating expression of gene in fat metabolism and IMF deposition in the quadriceps femoris of mice. However, it need to be further clarified that the specific signaling pathways, transcription factors and response elements of IMF deposition-related genes expression by dietary CLA in animals.

## Conclusion

In summary, in cellular level, our result demonstrates that CLA treatments significantly enhanced the activity of porcine PPARα promoter, and PPARα induced the expression of porcine A-FABP. Our results preliminarily revealed that CLA promotes the transcriptional expression of A-FABP through PPARα signaling pathway. In animal models, our study displays that dietary supplementation with CLA promotes fat deposition and increases IMF content by increasing the expression of PPARα and A-FABP. In addition, our current study also demonstrates that dietary CLA improves IMF content mainly by regulating the expression of IMF deposition-related genes. These results provide a theoretical basis for revealing the mechanism of CLA regulating IMF deposition.

## Data availability statement

The datasets presented in this study can be found in online repositories. The names of the repository/repositories and accession number(s) can be found in the article/Supplementary material.

## Ethics statement

The animal study was reviewed and approved by the Animal Care and Use Committee of Henan Agricultural University (HNND2020112613, approval date: 26/11/2020).

## Author contributions

GY and JC designed the study. RY and YL performed the experiments. JC conducted the statistical analysis and wrote the final version of the manuscript. GY assisted in the interpretation and revising of the article. HL checked the data. All authors have read and approved the final manuscript.
