# Supplementary material for: Conjugated linoleic acid regulates adipocyte fatty acid binding protein expression via peroxisome proliferator-activated receptor α signaling pathway and increases intramuscular fat content
Source: Front Nutr. 2022 Nov 29;9:1029864. doi: 10.3389/fnut.2022.1029864 (PMC9745092; doi:10.3389/fnut.2022.1029864)
Supplement: Supplementary file 1 [file Table_1.docx]

CLA diet composition is as follow.

Supplementary Table.1 Fatty acid profile of the conjugated linoleic acids (CLA) supplements^a^

| Fatty acid | CLA |
| --- | --- |
| C16:0 | 5.24 |
| C18:0 | 2.15 |
| C18:1 | 11.65 |
| Linoleic acid (LA) | 0.75 |
| cis-9,trans-11 CLA | 37.15 |
| trans-10,cis-12 CLA | 36.96 |
| Other CLA isomers | 5.57 |

^a^The percentage of compositions by weight.

Supplementary Table.2 Compostion and nutrient benefits of basal diets

| Items | Content | Component | Content |
| --- | --- | --- | --- |
| Chicken powder | 4.0 | Water | 96.0 |
| Soybean meal | 21.0 | Crude protein | 188.0 |
| Fish meal | 12.0 | Ether extract | 52.6 |
| Vegetable oil | 3.8 | Crude fibre | 30.6 |
| Bran | 10.0 | Crude ash | 54.9 |
| Corn | 40.0 | Lsyine | 14.0 |
| Flour | 6.5 | Cystine | 6.5 |
| Vitamin | 1.0 | P | 7.2 |
| Trace element | 0.5 | Ca | 11.2 |
| CaHPO_4_ | 1.2 | Methionine | 5.0 |

Comments：1)The premix is provided for full price per kilogram: Vitamin A 14 kIU, Vitamin D 1.5 kIU, Vitamin E 120 IU, Vitamin K 10 mg, Vitamin B1 13 mg, Vitamin B2 12 mg, Vitamin B6 14.4 mg, Niacin 120 mg, Pantothenic acid 48 mg, biotin 0.4 mg, folic acid 12 mg, choline chloride 2.4 g, copper 20 mg, iron 240 mg, zinc 30 mg, manganese 75 mg, iodine g 2.0 g, sodium 2.0 g, selenium 0.2 mg.

2) Crude protein, calcium, and phosphorus are all measured values, and the others are calculated values.
