# Supplementary figures and images for: Conjugated linoleic acid regulates adipocyte fatty acid binding protein expression via peroxisome proliferator-activated receptor α signaling pathway and increases intramuscular fat content
Source: Front Nutr. 2022 Nov 29;9:1029864. doi: 10.3389/fnut.2022.1029864 (PMC9745092; doi:10.3389/fnut.2022.1029864)

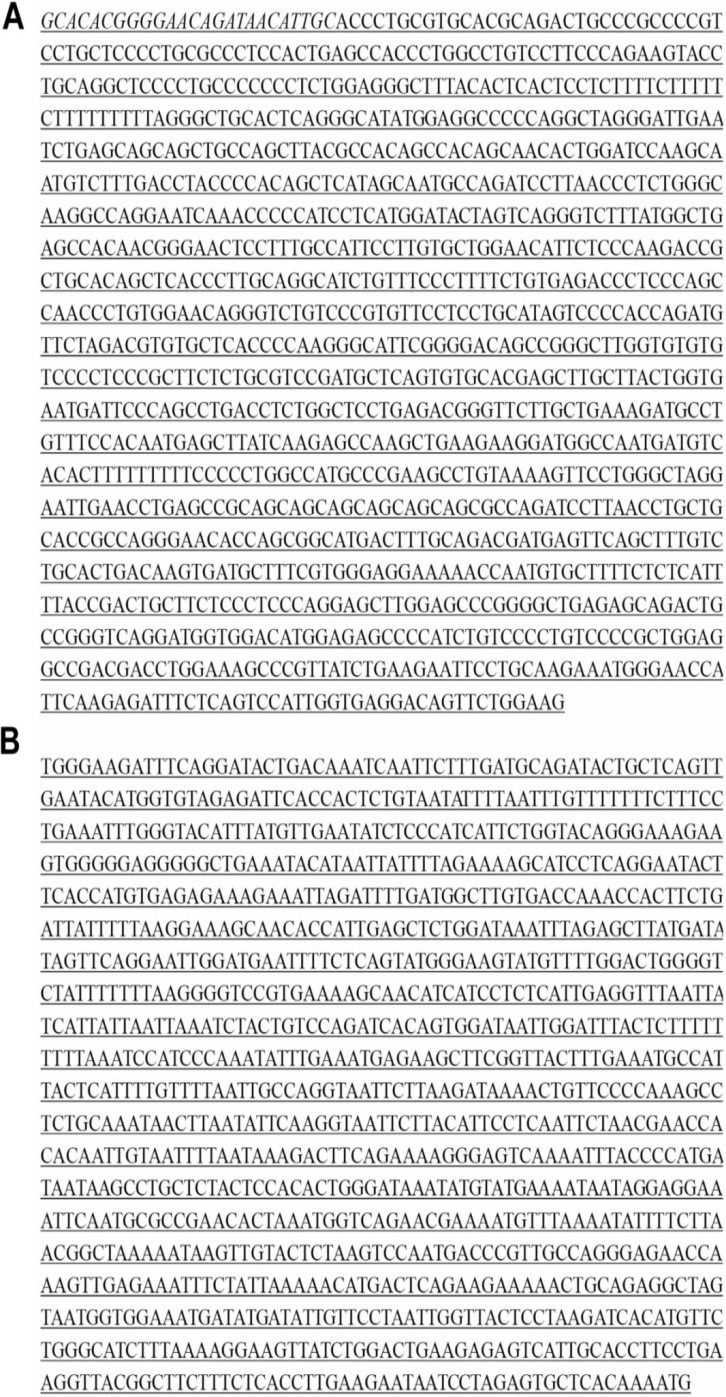

Supplement: Supplementary file 2 [file Image_1.JPEG]

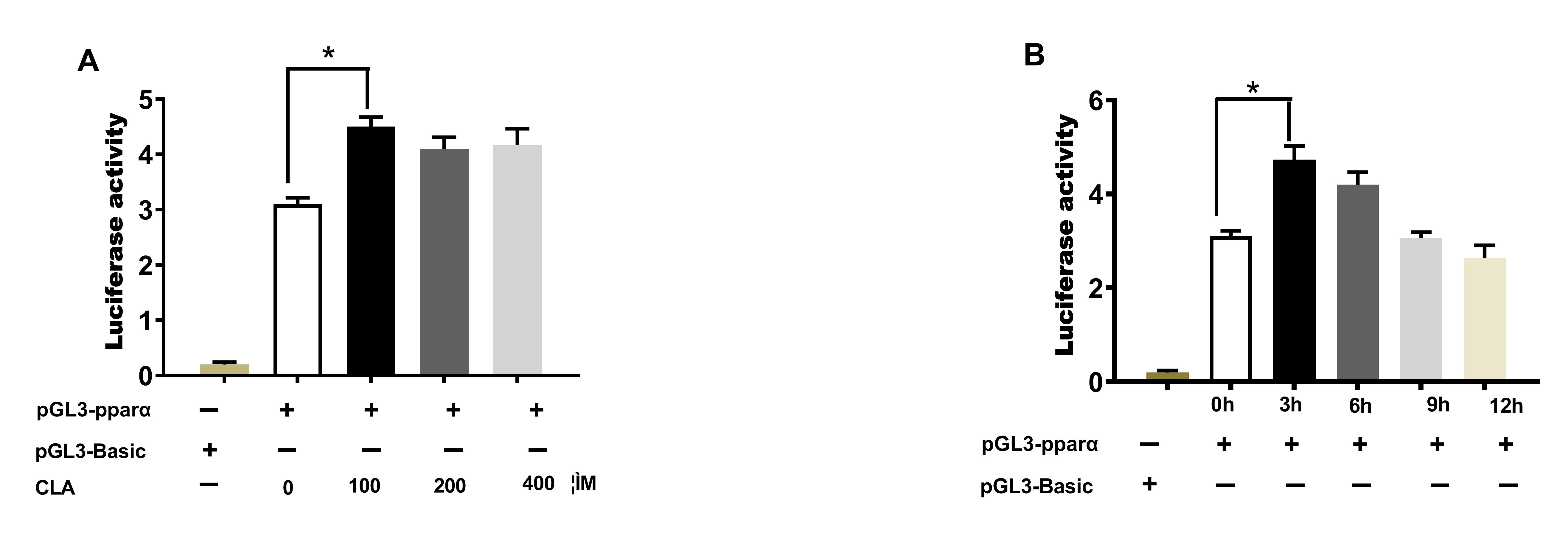

Supplement: Supplementary file 3 [file Image_2.JPEG]

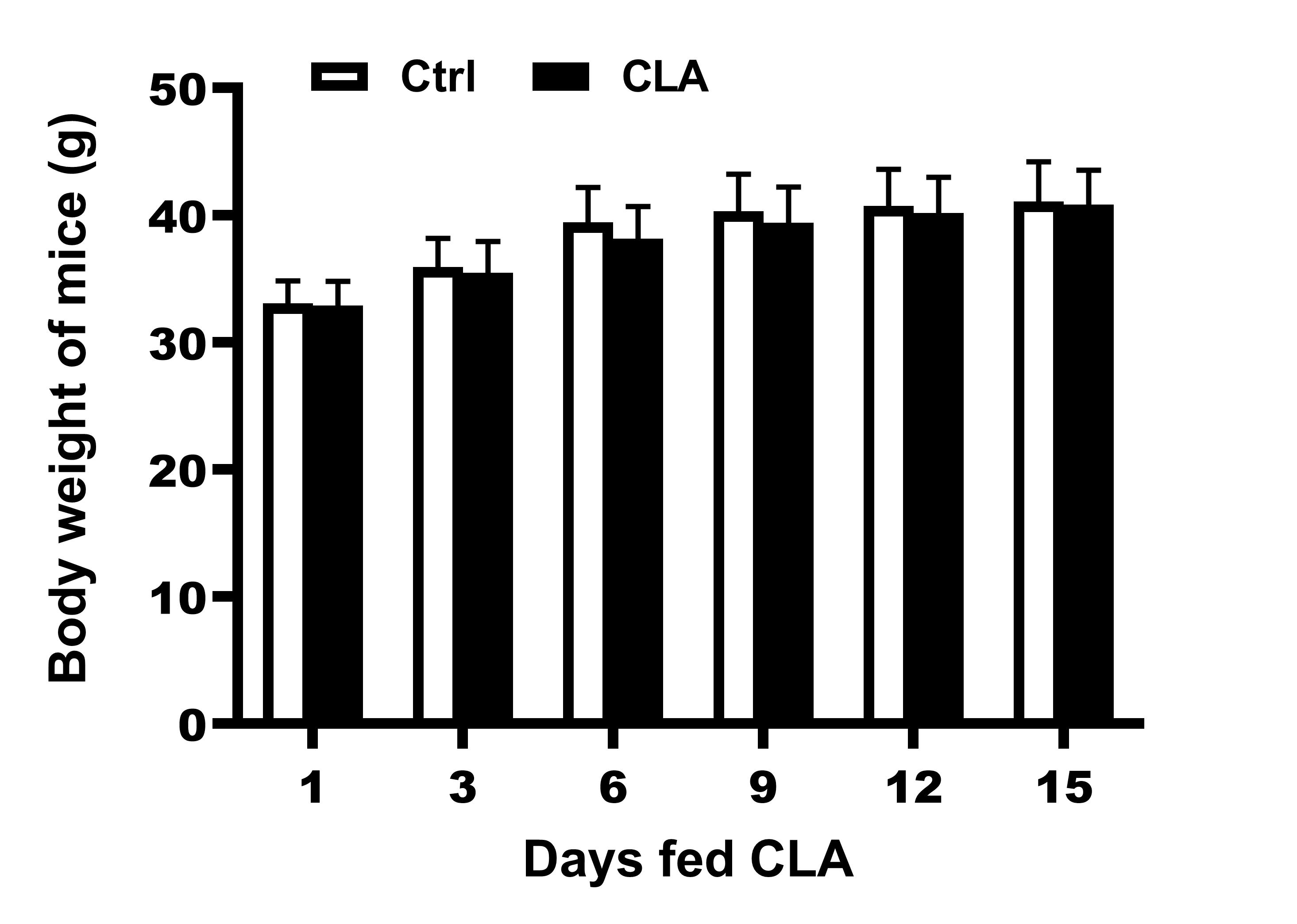

Supplement: Supplementary file 4 [file Image_3.JPEG]
